# Climbing the gap: a review on sex differences in high-level rock climbing

**DOI:** 10.3389/fspor.2025.1736831

**Published:** 2026-01-09

**Authors:** Kaja Langer, Andrea Roffler, Pauline Hief, Marie-Therese Fleddermann

**Affiliations:** 1 Department of Human Sciences, Institute of Sport Science, Technical University Darmstadt, Darmstadt, Germany; 2 Department of Movement Science and Training in Sports, Institute of Sport Sciences, Goethe University Frankfurt, Frankfurt am Main, Germany

**Keywords:** bouldering, elite, female, lead climbing, speed climbing

## Abstract

Since its inclusion in the Olympic Program in 2016, climbing has grown increasingly popular and professionalized. While climbing research is also increasing, female (elite) athletes remain underrepresented, with the extent of this gap remaining unexplored. Therefore, the aims of this review are to (1) systematically review the published research in climbing, focusing on the inclusion of female athletes, (2) evaluate the differences between male and female climbers, and (3) formulate recommendations for future research and practice. A systematic literature search was performed in four databases in July 2025. In a general analysis, all included studies were analyzed regarding the representation of female participants. Peer-reviewed original studies assessing various factors of climbing performance in advanced to higher-elite climbers (speed, lead, and boulder—IRCRA minimum of 15 females and 18 males) were included. In a detailed analysis, studies specifically assessing sex-specific differences were categorized and analyzed with respect to these differences. A total of 246 met the inclusion criteria and were included in the general analysis. The results showed that studies in high-level climbing predominantly focus on male participants, both in terms of study design, with much more studies investigating isolated male athletes compared to females, and in participant distribution with only 22.7% female participants compared to male participants (66.5%). In addition, only 34 studies including sex-specific analyses were identified. The results demonstrate that male and female performance in the various climbing disciplines depend on different factors to varying degrees and reveal further important differences between male and female athletes. Our findings emphasize the need for future climbing research to focus on female athletes and further investigate sex-specific differences. Based on our findings, we propose recommendations to guide future research and practice.

## 1 Introduction

Climbing has seen a rise in popularity among the general population, alongside a growing professionalization in competitive sport, particularly since its inclusion in the Olympic canon in 2016. An increasing number of studies in recent years have examined performance-related variables such as climbing-related strength and endurance (1–6), technique (7–9), and climbing-specific injuries (5, 10). Additionally, discipline-specific research across speed climbing, lead climbing, and bouldering has increased. While this research is crucial to understanding the notable physiological requirements of climbing performance (3, 11–14) and to providing the best possible support to athletes, it is equally important to recognize that certain groups of athletes remain significantly underrepresented in general as well as in discipline-specific climbing research: (elite) female climbers (3, 6, 13, 15). This gap reflects a broader trend in sports science, where female athletes are demonstrably less studied in many disciplines of exercise and sport science than male athletes (16–19), leading to the fact that research results are often not fully transferable to females. This is particularly relevant given the significant differences between males and females across various body systems, such as physiological or anatomic systems (4, 20, 21). For example, males were found to have larger muscle cross-sectional areas and a greater proportion of type II fibers compared to females (20). These differences lead to variations in motor performance such as increased strength, speed, or power output in males. In terms of climbing, these physiological and anatomical differences could influence performance results in bouldering, lead climbing, and speed climbing, leading, for example, to different adaptations in climbing strategy and technique between the sexes. However, data and studies are lacking, which leads to limited understanding and inaccurate generalizations regarding sex-specific performance in climbing. Addressing this issue would, therefore, provide a precise understanding of the basis on which evidence-based recommendations for females in climbing have been made. These insights may further contribute to future advancements in female athletic performance.

Several factors have been identified as relevant to climbing performance. Langer et al. (13) translated a conceptual model proposed by Weineck (22) to climbing performance and postulated, that climbing performance is influenced by a broad spectrum of interrelated factors. These include strength, endurance, speed, and flexibility; tactical and cognitive abilities; coordination and technical skills; psychological factors; anthropometry; and age. Beyond these determinants, nutrition, injury, and injury prevention, as well as the quality, structure, and periodization of training, have been found to be highly relevant to climbing performance and long-term performance outcome (3, 5, 6, 10, 23, 24). Furthermore, it has been proven that competition can influence performance (25), which is particularly important in lead climbing and bouldering, as the specific demands placed on athletes vary considerably from competition to competition and from route to route.

However, as noted above, research on differences between male and female climbing performance and related performance factors remains scarce. A recent review investigated this sex data gap in climbing and found only 36% of participants being females (26). Nevertheless, the extent of this gap in advanced to higher-elite climbing athletes has not yet been systematically investigated.

However, addressing it is crucial to gaining a clearer understanding of sex-specific differences in climbing performance, performance-related factors, and competition outcomes. This is especially important, since research on elite athletes is rare due to limited sample sizes, restricted access to participants, and the logistical challenges of elite sport environments, which may further reinforce the gap between the sexes (27). Key aspects that require attention include the representation of female athletes, the extent to which sex-specific analyses are conducted in the identified performance factors, and which differences have already been documented. Research in this field would not only contribute to a more comprehensive understanding of sex-specific climbing performance but also provide the basis for targeted training, recovery, and injury prevention for female athletes.

Therefore, the first objective of this review is to systematically review the published research on climbing (speed climbing, lead climbing, and bouldering), focusing on the inclusion of female athletes. Specifically, this involves identifying the proportions and distribution of male and female participants, as well as analyzing the number of studies that include only males, only females, or both sexes. In order to provide the best possible support for female athletes and their performance, the second objective of this review is to evaluate the differences between male and female climbers by conducting a detailed analysis of the studies including sex-specific analyses. The third objective of this review is to formulate recommendations for future research and practical applications based on the findings.

## 2 Methods

### 2.1 Search strategy and data sources

A systematic literature search was performed on PubMed, SURF, Web of Science (Core Collection), APAPsychInfo, and SPORTDiscus on July 25, 2025. The selection of databases was determined to cover a wide range of sport-specific disciplines. The literature search followed the Preferred Reporting Items for Systematic reviews and Meta-Analyses extension for Scoping Reviews (PRISMA-ScR) guidelines (28). Search-terms are presented in Supplementary Table S1. The free version of the program Rayyan (29) was used to facilitate and optimize the review process. The screening process was performed by two researchers who were blinded to the work of the other. After completing the screening process, remaining conflicts were resolved through discussion.

### 2.2 Inclusion and exclusion criteria

Studies were included into the general analysis if they (a) focused on climbing performance-related and determining factors, (b) included a representative sample of human lead climbers, speed climbers, and/or boulderers, and (c) included climbers based on the classification of Draper et al. (30). More precisely, we included participants with a minimum climbing level of 15 or 18 (advanced) on the International Rock Climbing Research Association (IRCRA) reporting scale (30) for females and males, respectively. Studies that also included participants below these performance levels were considered if their data were analyzed in relation to climbing level. The IRCRA scale was chosen to classify athletes' performance level because of its prominence in the climbing community and its internationally recognized framework for categorizing climbing ability. The IRCRA system provides standardized conversions across different national and discipline-specific grading scales, allowing for consistent comparison between studies that use different rating systems. Additionally, it allows for rating climbers irrespective of sex and age. The detailed analysis was based exclusively on the data relating to the included category. Youth athletes are frequently overlooked in climbing research, a limitation that has been highlighted repeatedly (31). Therefore, to fully cover professional climbing, studies including participants under the age of 18 were also included in the review. However, as it is not possible to directly compare young athletes with adults (31), these studies were analyzed in a separate category (Youth athletes). Only peer-reviewed original studies published in English or German were considered. Animal based research, as well as studies focusing on mountaineering and/or outdoor climbing were not included into this review. If full texts were not available online, the authors were contacted directly. If they were not able to share the full text within three weeks, the publication was excluded from this review. For the detailed analysis of sex-specific differences in advanced to higher-elite climbing, a subsample was extracted from the previously identified studies. This included all studies that had conducted a quantitative analysis of sex-specific differences in performance-related or performance-determining factors.

### 2.3 Data extraction and analysis

For the general analysis, the data extracted from all studies included participant characteristics (number, discipline, sex, age, and performance level). Specifically, we recorded the number of studies that focused exclusively on male or female participants, as well as those that included both sexes, and noted whether sex-specific analyses were conducted. The number of male and female participants were reported both in total and according to the climbing discipline. Furthermore, the distribution of participant sexes across the climbing disciplines was analyzed. To allow comparison of the results between studies, reported grades for climbing and bouldering performance were transformed according to the IRCRA scale (30). For the detailed analysis, studies that conducted sex-specific analyses were categorized according to performance-related and determining factors based on current literature (10, 13, 22, 25, 32) including energetically determined factors; flexibility; cognitive function and psychological abilities; coordination, skills, and technique; nutrition and energy availability; anthropometry; competition analysis; training and adaptations; injuries and mental health; and youth athletes. In addition, the methodological quality of the studies included in the detailed analysis was evaluated using the AXIS tool (33), a critical appraisal tool for cross-sectional studies. Each of the 20 AXIS items was rated as either positive (assigned a score of 1) or negative (assigned a score of 0). Three independent raters were assigned subsets of the identified studies so that each study was ultimately evaluated by two raters, resolving any discrepancies in a consensus meeting. Items 7, 13, and 14 were excluded from the evaluation, as all three items relate to experimental designs and were therefore not applicable to any of the included studies. Consequently, the maximum possible score was 17. Predefined percentage thresholds were used to categorize study quality, with scores ≥ 70% (≥12 points) indicating high quality, scores between 60.0% and 69.9% (10–11 points) indicating fair quality, and scores < 60% (<10 points) indicating low quality, following Rovito et al. (34).

## 3 Results

**Figure 1:**
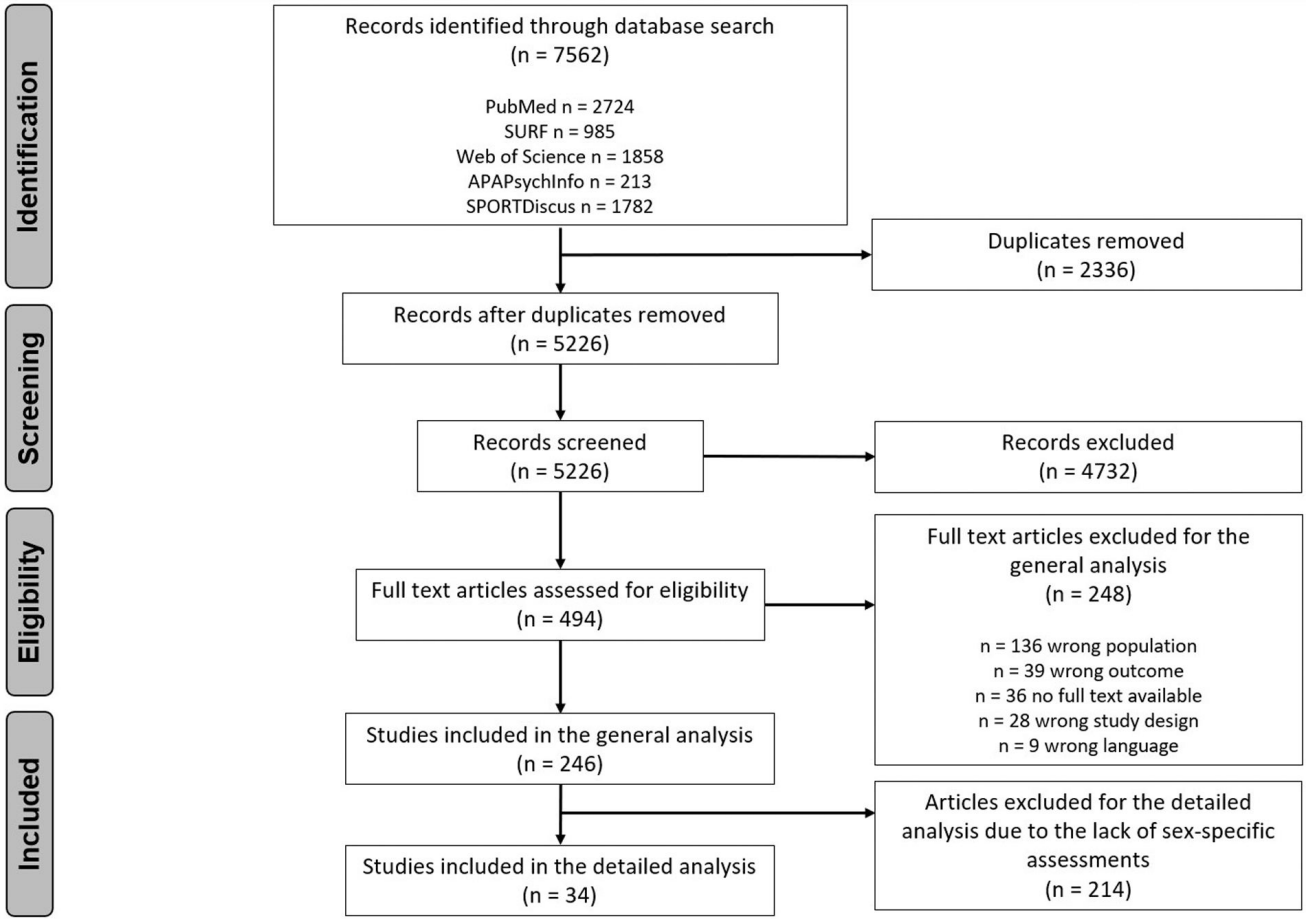
Preferred reporting items for systematic reviews and meta-analysis extension for scoping reviews (PRISMA-ScR) flow chart according to Tricco et al. (28).

The search of the databases provided a total of 7,562 references. After removing the duplicates, 5,226 references remained. Of these, 4,732 were excluded after reviewing the titles and abstracts. During a full-text screening of the remaining 494 references, a total of 248 studies were excluded for various reasons (see Figure 1). A total of 39 full-texts were not available even though all corresponding authors were contacted. A total of 246 studies met the inclusion criteria and were included in the general analysis. After screening the included articles for those assessing sex-specific differences, 34 studies were included into the detailed analysis (Figure 1).

### 3.1 General analysis

**Figure 2:**
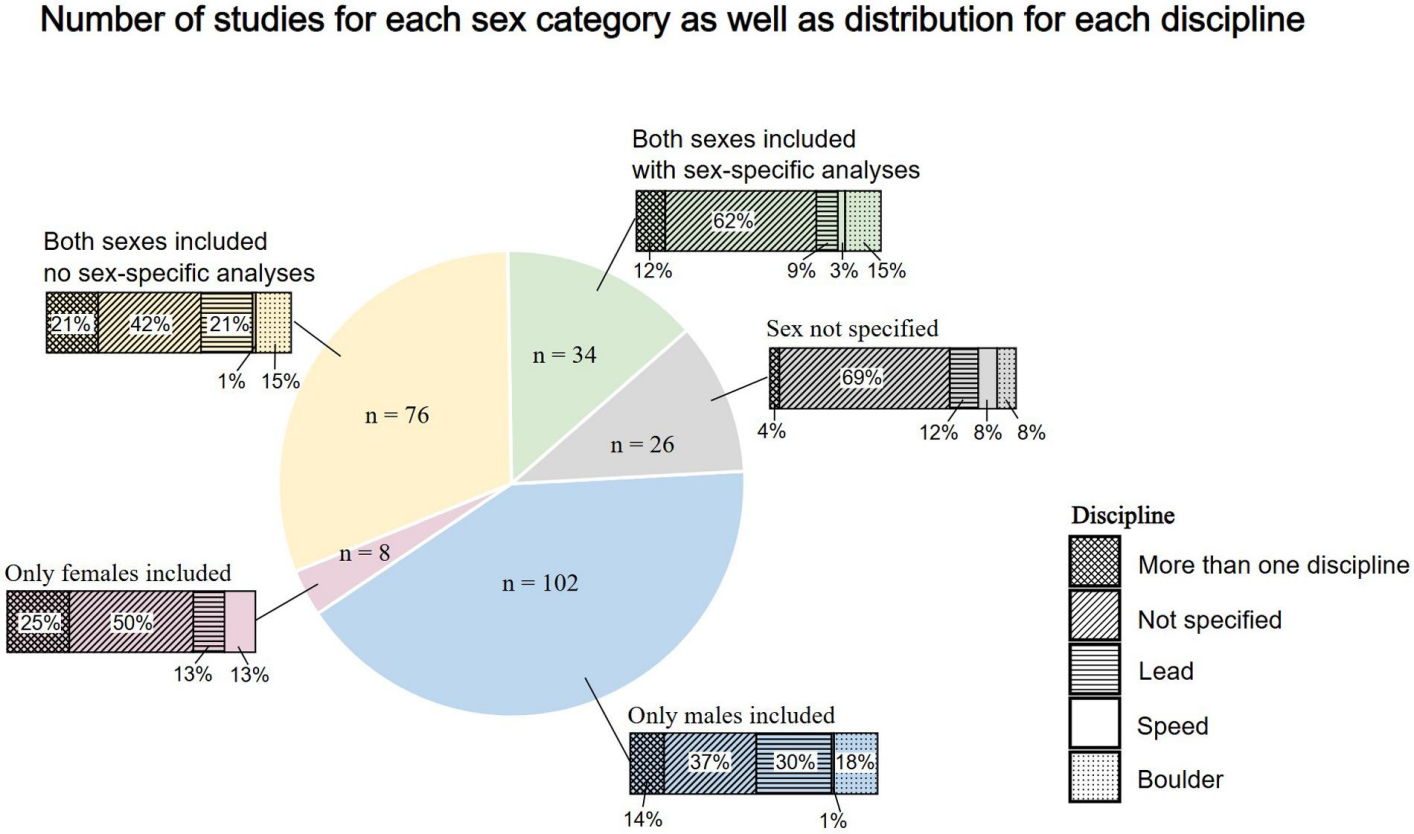
Number of studies regarding included sexes as well as distribution of specific discipline per sex category.

Of the 246 studies included in the general analysis, 102 studies included only male participants (41.5%) compared to 8 studies, which included only female participants (3.3%). 109 studies included both sexes, of which 34 studies performed sex-specific analyses (13.8%). In addition, 27 studies did not specify the sex of all or the majority of the participants (11.0%). Furthermore, looking at the distribution, 10,394 male participants (66.5%) were investigated compared to 3,555 female participants (22.7%). In 1,683 participants (10.8%), no further detail about the sexes were provided. Most studies (*n* = 113) did not specify climbing disciplines. Figure 2 shows the distribution of included sexes for each climbing discipline.

### 3.2 Detailed analysis

The 34 studies including sex-specific analyses were categorized into a total of ten categories based on the assessed performance-related and determining factors. As some studies addressed more than one factor, they were assigned to multiple categories. The categories included energetically determined factors (*n* = 8), flexibility (*n* = 3), cognitive function, and psychological abilities (*n* = 3), coordination, skills, and technique (*n* = 2), nutrition and energy availability (*n* = 8), anthropometry (*n* = 5), competition analysis (*n* = 6), training and adaptations (*n* = 2), and injuries and mental health (*n* = 2). Studies examining sex differences across various factors in elite national youth athletes (*n* = 5) were analyzed in a separate category. A summary of all studies included in the sex-specific analysis can be found in Supplementary Table S2. Overall, the study quality was good with only 28 out of 34 studies having a score >70% which is rated as high quality. Initial discrepancies between raters ranged from 13.2% to 26.2%. All disagreements were resolved during the consensus meeting. A summary of the study quality assessment can be found in Supplementary Table S3.

#### 3.2.1 Energetically determined factors

A total of eight studies assessed sex-related differences in terms of climbing-specific, energetically determined factors including strength, endurance, and speed. Upper-limb muscular endurance and finger-flexor maximum strength (1, 35–37), finger-flexor muscular endurance (1, 35, 36), core muscular endurance, upper-limb power (36, 37), upper-limb maximum strength, as well as upper- and whole body aerobic endurance (37) were found to be important factors associated with climbing performance in both males and females. Nevertheless, Draper et al. (36) found that finger-flexor muscular endurance and upper-limb power explained a higher proportion of performance variance in female climbers (65%) than in males (35%). Berta et al. (35), on the other hand, reported that a model including finger-flexor maximum strength and muscular endurance accounted for 58% and 54% of the variability in climbing ability for males and females, respectively, with maximum strength being the strongest predictor for both sexes. While multiple studies revealed greater absolute and relative finger-flexor strength for male compared to female climbers (1, 38, 39), Baláš et al. (1) showed that this difference decreases with increasing climbing level. In regard to finger-flexor muscular endurance, Philippe et al. (38) found male climbers to have a greater force-time integral during a continuous finger-flexor muscular endurance test compared to females. However, no significant differences were found between the sexes for the force-time integral during an intermittent test and the time until failure in both tests. Additionally, no differences in de- and re-oxygenation were found between males and females during intermittent testing. Looking at the different disciplines, Winkler et al. (40) found female bouldering performance to be primarily determined by upper-limb strength and power, whereas male performance was additionally determined by lower-limb power. Female lead climbing performance was found to be strongly associated with upper-limb strength and power as well as finger-flexor muscular endurance, while male performance was found to be strongly associated with both lower- and upper-limb power. Speed climbing performance was found to be strongly associated with lower-limb power and upper-limb-strength and power for both sexes (40). De la Cruz et al. (41) assessed upper- and lower-limb force-velocity profiles in speed climbers in pull-ups and squats. They found no significant differences between the sexes for any of the dependent variables including maximum and relative force, velocity, power, one repetition maximum, and the percentage of the one repetition maximum where peak power was expressed.

#### 3.2.2 Flexibility

MacKenzie et al. (37) found male but not female climbing performance to be significantly correlated to hamstring and lower-back flexibility. Similarly, Draper et al. (36) found that hip-flexibility can distinguish between different ability levels in male but not in female climbers. MacKenzie et al. (37) on the other hand found no correlation to performance for hip-flexibility in both sexes, while Winkler et al. (40) identified hip-flexibility as a key determinant of bouldering performance in females, whereas no such association was found for men, nor for either sex in lead or speed climbing.

#### 3.2.3 Anthropometry

A total of five studies assessed anthropometric differences between male and female climbers. Overall, males tend to be taller, heavier, and have more fat-free mass and arm volume compared to females of similar performance levels (38, 39, 42). While males showed no difference in anthropometric measures when comparing finalist with semi-finalists, female finalists were significantly shorter, lighter, had a smaller fat-free mass and lower arm volume than semi-finalist (39). Another study by MacKenzie et al. (37) found no significant correlation of anthropometric measures and climbing ability for either sex. Solely, arm span in males and ape index in females were associated with performance. Baláš et al. (1) also reported female climbers to have a higher body fat percentage than male climbers. However, when comparing males and females across different performance levels, they found these differences to be greater at lower levels of climbing performance and less pronounced at higher levels.

#### 3.2.4 Cognitive function and psychological abilities

One study on cognitive functions and two studies on psychological abilities have examined sex differences in climbers. Garrido-Palomino et al. (43) investigated the relationship between attention and self-reported climbing ability in 25 male and 10 female climbers, finding no significant differences in any attention task. Garrido-Palomino and Espana-Romero (44) examined emotional intelligence in 33 climbers (15f, 28 m) of varying expertise using two instruments (Mayer-Salovey-Caruso Emotional Intelligence Test and Schutte Self Report Emotional Intelligence Test). Sex differences were initially found in emotion regulation and emotion expression, with male climbers scoring higher than female climbers. However, the differences disappeared after adjusting for age. MacKenzie et al. (37) reported no significant correlation between self-confidence and climbing ability in either sexes. In addition, cognitive or somatic anxiety as well as self-confidence did not discriminate between different levels of climbing ability in either sex.

#### 3.2.5 Coordination, skills, and technique

Two studies were found to address sex-specific differences in coordination and skills in climbing. Winkler et al. (40) found that simultaneous coordination of limbs in dynamic moves improved model fit in speed climbing for female athletes, but not in lead climbing or bouldering, whereas for male athletes it improved model fit in lead climbing and bouldering, but not in speed climbing. MacKenzie et al. (37) found the time balancing on a ball on one foot until failure to correlate significantly with female but not male climbing ability. Additionally, neither hand-eye nor foot-eye spatial coordination showed a significant relationship to climbing ability in both sexes.

#### 3.2.6 Nutrition and energy availability

A total of eight studies evaluated sex differences in nutrition and energy availability in climbing athletes. Six studies explored the energy intake or availability as well as macronutrients with inconsistent results. Chmielewska and Regulska-Ilow (45) found that low energy availability (LEA) exists in all groups irrespective of competition level and sex but is more frequent in male and elite groups. For macronutrients they found a lower carbohydrate intake for male but no differences between sexes in fat and protein intake when adjusted for body weight. Four studies (46–49) found no difference in mean energy intake between sexes with the mean energy intake being not below target. Mondero et al. (42), also found energy intakes below target for only one female. Carbohydrate intake, however, was below target for all athletes and protein intake was below target for only females. Five studies also explored the intake of micronutrients in climbing athletes (42, 45–48). Only two studies found differences in micro nutrient intake between sexes with both studies indicating a lower potassium intake for male (45, 47). Mondero et al. (42) showed that females show a lower iron status in blood but no difference in nutritional iron intake. Two studies explored disordered eating via a questionnaire (42, 50). One study reported a low prevalence for energy deficiency (42), while also showing that four out of eleven females were at risk for low energy availability (LEA) (42). One study reported a higher prevalence of disordered eating for females with half of females in the higher climbing levels showing disordered eating (50). Two studies compared supplement use between sexes (51, 52). Gibson-Smith et al. (52) found no difference in supplement use and knowledge between sexes. However, Chmielewska and Regulska-Ilow (51) showed that females showed different reasons for supplementation. For example, males reported more frequently to supplement for muscle maintenance.

#### 3.2.7 Competition

Overall, six studies examined sex differences in competitive climbing. In bouldering, Medernach et al. (53) examined the movement demands (number of attempts, attempt duration, time, rest time, and gripping time) and found that female athletes used a significant higher number of attempts and had a significant longer attempt duration. During World Cup finals in lead climbing, Arbulu et al. (54) also found that females, on average, spent longer in contact with holds and rested for significantly longer periods than males. Assessing the rest times between boulders during competition, La Torre et al. (55) found a slight, steady increase in resting heart rate in females, whereas no such change was observed in males. Künzell et al. (56) examined strategy changes in bouldering and found that the success rate of new solutions (visibly different from previous attempts) was substantially higher than that of repeated solutions, with males changing their strategy after failure in approximately 30% of attempts, compared to around 37% for females. While Winkler et al. (57) reported that the difficulty of lead climbing routes for females was at an IRCRA level of 25 in the qualification rounds and 26–27 in the finals, the routes for males were at level 26–27 in qualifications and 28 in the finals. Augste et al. (58) found no significant differences in the boulder types occurring in male and female bouldering. However, in Augste et al. (58) male athletes were found to be able to solve significantly more boulder problems that include dynamic and mantle movements than females. Female athletes, on the other hand, were found to perform better on slab problems. In addition, lower-ranked female athletes were found to perform significantly worse than those in the top 20 in the dynamo, volume, and crimp categories. In contrast, lower-ranked males performed worse in the dynamo and slab categories. Winkler et al. (57) found that male speed climbers had significantly shorter starting times, fewer upper- and lower-limb actions, and shorter contact and reach times for both upper and lower limbs compared to females. Additionally, female climbers were observed to use the crimp grip much more frequently than males.

#### 3.2.8 Training and adaptations

Sex-related differences in training responses and adaptations were assessed in only two studies. Baláš et al. (1) were able to show that both males and females achieve their highest levels of difficulty after seven to ten years of climbing. Higher climbing performance was associated with climbing-specific training in both sexes. In addition, MacKenzie et al. (37) were able to show that targeted training of upper-limb maximum strength and upper-limb muscular endurance in males and females, respectively, led to an improved climbing ability. Training of balance in males and lower-limb and core muscular endurance in females, however, did not lead to an improved climbing ability.

#### 3.2.9 Injuries and mental health

Grønhaug et al. (59) assessed chronic climbing injuries in intermediate to higher elite climbers. They identified no significant differences between sexes in regard to the rate of injuries. However, their analysis revealed that female climbers sustained significantly more injuries to the shoulders, neck, and head, whereas male climbers were significantly more often affected in the fingers, elbows, and ankles. Identeg et al. (60) assessed mental health in advanced to higher elite climbers compared to elite athletes from other sports. While climbers were found to express a slightly but not significantly higher prevalence of moderate to severe symptoms of depression, a slightly higher moderate to severe levels of stress, and a lower prevalence of severe symptoms of anxiety, no significant differences were found between males and females or between different performance levels among climbers.

#### 3.2.10 Youth athletes

Five studies examined sex differences in the anthropometry of youth climbers, while one study assessed nutritional differences. Vrdoljak et al. (61) assessed anthropometric differences (body mass, height, body mass index, arm span, ape index, and body fat percentage) between male and female youth climbers. They found that males had a lower body fat percentage and were significantly taller compared to the females. When comparing the height of the study participants with age-matched males and females, climbers tend to be smaller than their peers especially for males (25th percentile for females,15th percentile for males). No other differences between the sexes were found. Watts et al. (62) examined the anthropometric characteristics of young competitive climbers compared with physically active non-climbing children. They found the young climbers to present similar anthropometric characteristics to elite adult climbers, including shorter stature, lower body mass, and reduced skinfold thickness. Compared with age-matched non-climbers, they exhibited a more linear body shape with narrower shoulders relative to hips and a lower body fat percentage, despite having a similar body mass index. In addition, sex differences were observed, with girls showing significantly higher skinfold measurements at the triceps, thigh, and calf than boys. Gilic and Vrdoljak (63) assessed body mass, body height, body fat percentage, ape index, and finger-flexor maximum strength in youth advanced to elite climbers of two different age groups. They found no significant differences for any of the variables between both age groups. Comparing boys and girls, they found that body weight and height significantly correlated with age and maturity offset in boys, while finger-flexor maximum strength in the sitting position significantly correlated with maturity offset in girls. Baláš et al. (64) assessed anthropometric and strength parameters in youth and adult advanced to elite climbers. Despite similar climbing performance levels and no difference in meters climbed per week, body mass and height were found to be significantly greater in adults and significantly higher in males. The ratio of extra cellular and body cellular mass as well as body fat percentage was found to be greater in adults and greater in youth female climbers. Furthermore, finger-flexor maximum strength was found to be higher in youth male climbers within both age groups but did not differ between the age groups. Finger-flexor muscular endurance was found to be higher in youth climbers but did not differ between the sexes. Upper-limb muscular endurance, however, did not differ between age groups or sexes. Schöffl et al. (65) found that male youth climbers had higher performance levels and a greater ape index than females, while females had lower weight, BMI, and leptin levels compared to non-climbing peers; both sexes exhibited lower skinfold thickness, but only youth males had lower fat mass. For nutrition Michael et al. (23, 24) found no significant differences between boys and girls in energy and macronutrient intake. However, as youth males have a higher energy requirement, energy intake, carbohydrate intake, and fat intake were significantly below target only in youth males. The scores for eating disorder were relatively low and showed no differences between sexes.

## 4 Discussion

The purpose of this review was twofold. The first aim was to assess the extent of the sex data gap in climbing research. As demonstrated in previous research, female athletes have been far less studied across all areas of exercise and sport science compared to their male counterparts (19). The findings of this review confirm that this sex imbalance is also evident in climbing research, highlighting a clear male dominance. The second aim was to analyze sex-specific differences observed in advanced to elite male and female climbing athletes. Findings from only 34 studies included in the detailed analysis demonstrate a lack of sex-specific analyses in climbing research. Nevertheless, sex-specific differences have been identified across various performance relevant and determining factors, highlighting the relevance of research in this field.

### 4.1 Sex data gap in climbing research

Overall, our findings show that climbing remains generally under-researched, especially at the high-performance level. We found only 246 studies, which examined elite or advanced climbers [defined according to Draper et al. (30) as having an IRCRA level of 15 or higher for females and 18 or higher for males]. In addition, most studies failed to specify the climbing discipline, which limits the contextual interpretation of research findings.

When examining the sex-comparison, the majority of high-performance climbing studies focus exclusively on male climbers, while research dedicated solely to female athletes remains scarce with a ratio of 41.5% (male) to just 3.3% (female). In absolute numbers, 102 studies were conducted exclusively with male climbers, whereas only 8 studies focused solely on female climbers. Additionally, 27 studies did not specify the sex of their participants and 109 studies included both sexes. Interestingly, however, even in mixed-sex studies, the proportion of male climbers was very high (70%). One fifth of the studies included fewer than 20% female athletes, with many including only one or two female participants. Moreover, only 34 (31.2% of all studies including both sexes) performed sex-specific analyses. However, such analyses are crucial not only to identifying sex-specific differences but also for preventing biased conclusions and for developing evidence-based recommendations that are effective for both sexes, especially for female athletes, who remain underrepresented. Therefore, to gain a clearer picture of this sex data gap, we also examined the overall number of participants across all studies with the result of an evident male bias: 66.5% of participants were male, compared to only 22.7% female, while the sex of 10.8% of the participants was not reported. However, it is reasonable to assume that these were also male participants, given the historical standard of participants being male. If we assume the unknown participants were female, the representation of female athletes would still only reach 33.5%. Overall, these findings are in line with previous literature addressing the sex data gap in different areas of sport and exercise science. For example, in their review Costello et al. (16) demonstrate that female participants are in general underrepresented in sport and exercise science. They analyzed the sex distribution of participants in three leading sport and exercise science journals between 2011 and 2013 and found that females comprised only 39% of total participants. Building on this, Cowley et al. (17) updated these findings, reporting that females accounted for only 34% of total participants and just 6% of total publications between 2014 and 2020 in six leading sport exercise science journals. A recent review in climbing (26) showed similar findings than Costello et al. (16) and Cowley et al. (17). They investigated female representation in rock climbing irrespective of performance level and found a participation rate of 35% females in their reviewed studies. In contrast, we observed a much lower participation rate of females (22.7%), which could be explained by our focus on advanced to higher-elite athletes. Therefore, the imbalance regarding the sex of participants appears to be even more pronounced among advanced to higher-elite athletes. In addition to that, Lee et al. (26) excluded studies that did not report participant sex. Even when recalculating according to their criteria, female representation in our dataset remained low at 25%. Regarding study types, we observed a higher prevalence of male-only studies (41.5%) compared to female-only studies (3.3%). When excluding studies with unreported sex, the prevalence of male-only studies increased to 46.6%, while female-only studies accounted for only 3.7%. This may be because female climbers are particularly well represented at lower to intermediate levels, which could be due to the fact that females have only recently begun participating in the sport in greater numbers (66, 67). Additionally, advanced to higher-elite athletes constitute a highly selective group. Therefore, especially in female athletes, studies often remain underpowered (68), which, according to Burden et al. (68), may lead to fewer studies involving female participants being published.

Furthermore, interestingly, the proportional distribution of studies including female participants in climbing is lower than in the studies of Costello et al. (16) or Cowley et al. (17), with only 22.7% female participants in climbing research compared to 34% and 39% in general exercise science research. This becomes especially relevant considering that with 69% the majority of studies in the field of high-performance climbing have been published after the year 2014. At this time, the sex data gap was already well-known and documented in the scientific community for example by Costello et al. (16). In addition, according to the Olympic charter (in 2013), one responsibility of the international Olympic committee is “to encourage and support the promotion of women in sport at all levels and in all structures, with a view to implementing the principle of equality of men and women” (69). This raises the question of why such a strong male bias still persists in elite climbing research, despite supposedly comparable structural conditions for males and females in sport (69).

One possible explanation could be a systemic and methodological bias in research, shaped by historical trajectories. Traditionally, it was assumed that physiological responses on exercise did not significantly differ between male and female athletes. As a result, male participants were (incorrectly) considered as “standard”. They were therefore predominantly chosen as study participants and findings were generalized to female athletes (17). However, “women are not simply small men” (70) and differ physiologically and anatomically from males, which is why research results based on male participants cannot be directly transferred to female athletes.

A second possible explanation is introduced by Paul et al. (18). The authors examined the sex distribution across various sports such as soccer, basketball, rugby, or baseball/softball, conforming a clear under-representation of female athletes. They argued that elite sports are often male dominated, which has resulted in males being predominantly studied in this field as well. Furthermore, financial considerations and the generally financial support for female sports have been argued as another factor. When applying these arguments to climbing and to our findings these explanations may, however, not be applicable. As noted earlier, climbing is a relatively young sport, introduced to the Olympic program only in 2016. It therefore seems unlikely that male-dominated structures have influenced elite climbing to a degree sufficient to account for the observed imbalance in research. Nevertheless, even though the number of females in climbing has been increasing (66, 67), a male dominance still exists in sub-elite climbing (71, 72), which may have a similar effect on climbing research.

Another commonly discussed reason for the under-representation of female participants in sport and exercise science literature is the composition of the research team (73). Specifically, studies with predominantly male first-authorship tend to include much less female participants. Therefore, we performed a *post-hoc* analysis examining the probability of including female participants based on the gender of the first author and the year of publication (Supplementary Figure S1). Our findings revealed that when a female researcher is listed as the first author, the likelihood of including female participants increases by a factor of 3.7. This is consistent with the findings of Lee et al. (26), who reported that studies with female first authors had significantly higher proportions of female participants than those with male first authors. Moreover, regardless of the corresponding author's gender, the likelihood of including female participants has increased over time by approximately 4.2% per year.

Finally, the under-representation of females in climbing becomes even more dramatic when comparing the performance of male and female athletes. Compared to other sports such as the 100 m dash, the performance gap between males and females in climbing is remarkably small. According to World Athletics (74), the fastest woman since 1,900 is slower than the 8,139 fastest men in the 100 m dash, highlighting a large sex-based difference in sprint performance. In these kinds of sports, the mechanisms underlying performance differences are based on sex hormones (e. g. testosterone) as well as anatomical differences such as muscle mass or muscle cross-sectional area (20). In contrast, climbing shows much smaller disparities. Although speed climbing, which most closely resembles the demands of sprinting, still exhibits considerable sex differences (75), the gap narrows substantially in lead climbing and bouldering. According to athletes' reports, the hardest route climbed by males is graded 9c on the french sport scale (achieved by three climbers), while the hardest route climbed by a female is only one grade lower (9b+). These smaller performance differences between the sexes compared to many other sports, sex-specific analysis may provide insights into how technique, tactics, and strength are equally important, rather than muscle mass alone.

### 4.2 Sex differences in climbing

Climbing performance has been proven to be influenced by a broad spectrum of interrelated performance-determining and performance-relevant factors (3, 5, 6, 10, 23, 24). In this review, 34 studies included sex-specific analyses of various factors. While many studies analyzed sex-specific differences regarding anthropometry, strength, endurance, speed, flexibility, and nutrition and energy availability, research remains limited regarding sex-specific differences related to coordination, skills, and technique, injuries and mental health, cognitive function and psychological abilities, and training and adaptations.

Our findings emphasize that the performance of male and female climbers in different disciplines depends on different factors to varying degrees. While female lead climbing performance was found to be particularly influenced by upper-limb strength and power, as well as finger-flexor muscular endurance, male lead climbing performance, on the other hand, appeared to depend primarily on upper- and lower-limb power, upper-limb strength and endurance, as well as coordination. Furthermore, our results suggest that lower-level male lead climbing performance is additionally influenced by hip, lower-back, and hamstring flexibility, whereas at the elite level, hip-flexibility seems to be especially relevant for female bouldering performance. In addition, both male and female bouldering performance were found to depend on upper-limb strength and power, as well as finger-flexor muscular endurance, whereas male bouldering performance was additionally influenced by coordination. In speed climbing, performance in both sexes was primarily determined by lower- and upper-limb power, as well as upper-limb maximum strength. In males, performance was additionally influenced by upper-limb muscular endurance, whereas in females, it was further explained by finger strength and coordination. In addition to differing factor weightings between male and female athletes across disciplines, these findings show that especially male lead climbing performance seems to depend on a broader spectrum of factors than female performance. This is supported by findings from MacKenzie et al. (37), who reported that 23 out of 47 tested variables were found to correlate with climbing performance in males, whereas in females only 10 out of 47 variables showed significant correlations.

While both male and female climbing performance was found to benefit from a large arm span or ape index, multiple studies have shown that male climbers are, on average, taller and heavier than female climbers. This aligns with the findings of Hunter and Senefeld (20), who reported that, in general, males have longer and denser bones, as well as larger muscle cross-sectional areas, contributing to their greater height and body mass. Additionally, males have been proven to have larger, faster, and stronger muscles (20). Furthermore, the findings are consistent with general sex differences indicating that females tend to be more flexible than males, due to lower tendon stiffness (76, 77) and smaller muscle volume (20) in females. Nonetheless, while female climbers have been found to have a higher body fat percentage, a trend also observed in the general population (20), this difference was found to decrease with increasing performance levels. This is, no surprise in a sport that relies on a high strength-to-mass ratio (21). Nevertheless, it shows that especially female elite athletes show extremely low body fat percentages.

These findings are consistent with research on energy availability in climbing. While some studies reported that participants' total energy intake met target values, others found insufficient carbohydrate intake in both sexes and inadequate protein intake in females. Moreover, it should be noted that females, possibly due to menstrual cycle-related factors (78), have lower blood iron levels which can also impact performance (79). Supplementation did not vary between sexes, however, the reason for supplementation tended to differ with males supplementing more often for increasing muscle mass and performance (51). It is notable, that climbers of both sexes rarely relied on climbing-specific supplements and that only a low portion of climbers had access to nutritional support. These findings emphasize the potential risk of LEA and RED-S in climbing and align with the findings of Tan and Schöffl (80).

Other performance-related factors in sports climbing include cognitive and psychological variables, which are rarely examined in elite female climbers. Our findings show that only three studies met our inclusion criteria in this field. The findings suggest that psychological and cognitive factors are not determined by sex but are more likely influenced by individual differences such as training experiences or personal trait factors. However, it must be mentioned, that the distribution of participants also revealed a clear sex-imbalance, which may have influenced the results. For example, in the study by Garrido-Palomnino et al. (43), only 10 female climbers compared to 25 male climbers were examined. Therefore, these findings must be interpreted with caution. Furthermore, our findings align with those of a recent review by Mangan et al. (81), which investigated psychological factors in rock climbers. They also reported an underrepresentation of female participants and a lack of sex-specific analyses, which limited the interpretability of the results.

Another potential factor that could explain our findings is the difference in route setting between male and female competitors. As our findings show, there are differences between the competition boulders for males and females. While these differences could explain physiological differences between male and female climbers, they may also account for the reported sex-specific injury-patterns (59). Therefore, our results clearly show that differences between male and female climbers should also be considered in injury prevention. While a recent review on injuries and injury prevention in climbing (5) provided general training recommendations for injury prevention, it did not include any sex-specific guidance. Our findings, however, indicate that both climbing performance and injury prevention, given the differing injury patterns, may benefit from sex-specific and discipline-oriented training approaches. Considering the identified performance-determining factors, current research findings point towards incorporating various elements into climbing training. This is especially due to the fact that while research on sex differences in resistance training has shown that males and females adapt similarly to hypertrophy training (82), females were observed to demonstrate greater effect sizes in upper-body strength than males (82), which points towards potential sex-related differences in the outcomes of climbing-specific strength training.

#### 4.2.1 Youth athletes

Similarly to male adult climbers and the general population, boys were found to have lower body fat percentage than the girls. However, especially boys were found to show low carbohydrate and fat intake. In addition, boys were found to be taller and heavier, which significantly correlated with age and maturity offset. Finger-flexor maximum strength, on the other hand, correlated with maturity offset in girls, which might be related to an earlier maturation in girls (63). Alternatively, it may be related to a specificity of sports selection and sampling (83).

None of the studies that fulfilled our inclusion criteria assessed sex-specific differences regarding other performance determining factors in youth climbing. A first study by Nichols et al. (84), however, indicates that performance determinants in youth climbers are generally very similar to those in adults, encompassing primarily upper-limb power, maximum strength, and muscular endurance, as well as finger-flexor maximum strength and endurance.

### 4.3 Integrative summary

Taken together, the findings of this review highlight that sex-specific differences exist across all performance-determining and performance-relevant factors in climbing. While several determinants overlap between male and female athletes, their relative weighting differs between sexes and climbing disciplines. Although we addressed these factors individually, it must be emphasized that sport performance results from a complex interplay of multiple interacting factors (85). This underscores the importance of considering athletes as multifaceted individuals rather than isolating single determinants. However, research on sex-specific differences in climbing remains limited, and the interactions between factors such as strength, endurance, coordination, flexibility, psychological characteristics, and nutritional status are still insufficiently investigated.

## 5 Recommendations

**Figure 3:**
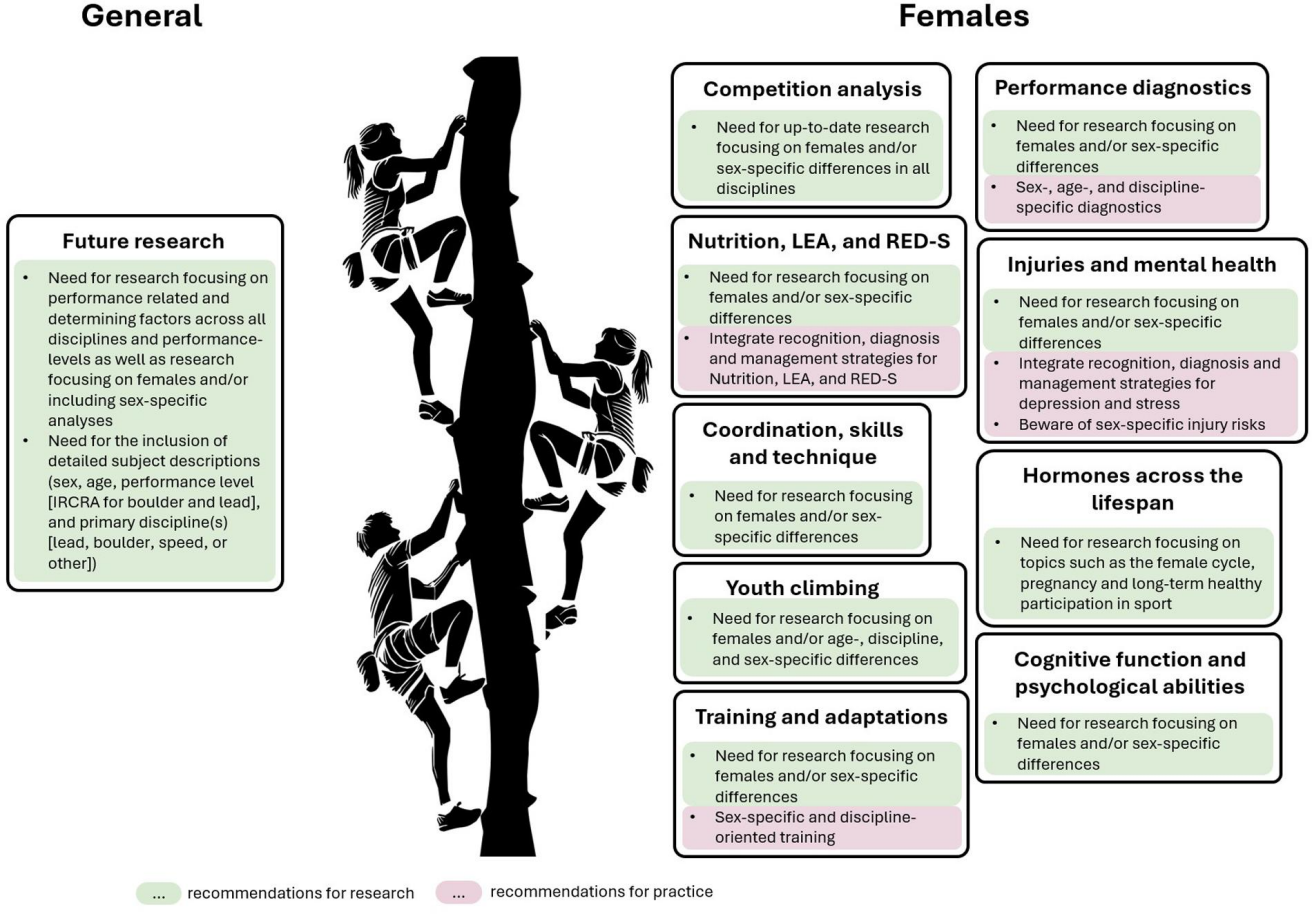
Recommendations for future research and practice.

In recent years, there has been a clear trend towards increasing participation by females in sport in general, which is also true for climbing. For example, at the 2024 Olympic Games, male and female climbers each accounted for 50% of participants, demonstrating sex disparity. Further, also in national and international climbing events there is a growing participation of female athletes. Despite this trend, research, and often its practical application, lags behind. Based on our findings and considering the limitations of this review, we propose several recommendations for both research and practice (Figure 3).

Firstly, we would like to highlight that both climbing research and practice would benefit greatly if the characteristics of the subjects were explicitly stated in published papers. Besides detailed information on participants' sex, this includes climbing performance level [best indicated using the IRCRA scale (30)]; age, and a more precise definition of the subjects' discipline(s). This would allow researchers as well as practitioners to gain a deeper overview and contextualization.

While high-performance climbing is greatly under-researched and the whole field would benefit from further analyses of climbing performance-related and determining factors, our results clearly demonstrate that especially females are significantly underrepresented in all areas of climbing research. This highlights the urgent need for further research in this field to specifically address female athletes. Studies that explicitly focus on female athletes or analyze sex-specific differences would be particularly helpful in this regard.

This includes future research on coordination, skills, and technique to determine if and how required and inherent skill sets in climbing may differ between sexes and thereby support the development of tailored training programs. In addition, future research should address injuries and mental health, aiming to clarify how males and females may need to train differently to prevent injuries and address mental health challenges.

Regarding cognitive function and psychological abilities, our results indicate an urgent need for more data in this field. This is also confirmed by a systematic review (81), which reported a lack of research involving female climbers. Key psychological and cognitive factors in performance climbing appear to be flow, confidence, anxiety facilitation, and perception (81), as well as decision making, problem solving (86), and route reading (87, 88). However, since these findings are primarily based on male participants, it remains to be determined which of these key factors also apply to female climbers. Detecting these factors helps to improve the performance of female athletes in climbing.

Another area within climbing that would particularly benefit from future studies focusing on sex-specific differences is the investigation of training effects and adaptations, as our results suggest potential advantages of sex-specific, discipline-oriented training. Of particular interest are potential differences in the timing and extent of adaptations to various training interventions, such as finger-flexor muscular endurance training or upper- and lower-limb power training.

Furthermore, up-to-date competition analyses across all disciplines would help to better understand the differences between performance determining factors of male and female climbing, as well as any possible changes to these profiles over time and the impact of route setting. Furthermore, the different weighting of the determinants of male and female climbing performance across the disciplines, as well as a different physiology between youth and adult athletes, implies a need for sex-, age- and discipline-specific performance diagnostics. To account for potential differences, test validation should include both female and male participants, and correlations between test results and climbing performance should be analyzed separately by sex. In addition, the diagnostics must account for potential changes in the demands placed on the athletes over time and must be kept up to date, which again emphasizes the need for up-to-date competition analyses. We therefore urge all researchers to pay closer attention to female climbers in future studies.

In addition, more research is needed in the field of youth climbing to identify the key factors influencing performance and development such as strength, technique as well as cognitive or psychological skills. Especially sex differences in maturation and their effects on climbing performance, as well as youth competition analysis play an important role in this field. Research in these areas is necessary to elucidate the performance-determining factors that differentiate young athletes from adult athletes. As shown in the discussion, there is no study on an elite level addressing these aspects in a youth population. Additionally, investigating differences between sexes and disciplines will help to develop effective age-, maturation- and sex-specific training and diagnostic strategies, optimizing skill development, and foster long-term athlete development.

As in any sport, climbing training should be individually tailored to the specific needs and characteristics of each athlete. This includes considering both discipline-specific and sex-specific performance demands and injury risks. A summary of the factors found to be especially important for each discipline and sex is presented in Table 1. The table is tailored to the differences between the sexes and does not intend to suggest that only the listed elements should be considered when planning a climbing training program. In addition, as research on the effects of training on climbing performance in general, and on female athletes in particular, remains limited (3, 6), the table does not provide specific training recommendations but rather presents a compilation of components that may be important for sex-specific climbing training.

**Table 1:** Summary of the factors possibly important for sex-specific and discipline-oriented climbing training.

Discipline	Male	Female
Speed	Lower-limb power Upper-limb power Upper-limb maximum strength Upper-limb muscular endurance Coordination	Lower-limb power Upper-limb power Upper-limb maximum strength Finger-flexor maximum strength Coordination
Boulder	Coordination Include a large movement repertoire Encourage route exploration Especially include dynamic boulders and slabs	Intermittent muscular endurance training to improve recovery between multiple attempts Coordination Hip-flexibility Especially include dynamic and strength-oriented routes
Lead	Upper-limb power Lower-limb power Coordination Lower level: hip-, lower-back and hamstring flexibility	Finger-flexor muscular endurance Upper-limb maximum strength Upper-limb power Coordination

In addition, coaches should be aware that, regardless of sex, climbers are at a higher risk of depression and stress than athletes from other sports, and should therefore integrate respective diagnosis, and management strategies into the training practice. Finally, our findings indicate that climbers and particularly high-level female climbers, express very low body fat percentages. This is no surprise, as a high strength-to-mass ratio is beneficial to climbing performance. However, low body fat percentage has been identified as an important risk factor for low bone mass, increased bone loss (89), LEA, and relative energy deficiency in sport (RED-S) including a potential danger to various important body functions (23, 24). According to our findings, both male and female climbers are in fact at a high risk of LEA and RED-S. Coaches should therefore integrate respective recognition, monitoring, and management strategies into the training practice. Especially RED-S should be considered an important topic that requires further research in climbing as it is gaining increasing attention, particularly in females. This is also closely related to the female hormonal cycle. In this context, recent studies emphasize the importance of the female hormonal cycle, e.g. related symptoms (90–92). Since this review focused on studies including male and female athletes, we did not include analyses regarding the female hormonal cycle. However, it is highly recommended that further studies should consider hormonal fluctuations across the whole lifespan including puberty and maturation, as well as possible pregnancy (93). Given that pregnancy does not mark the end of an athletic career, its necessary to develop clear criteria and guidelines for this special group. At the moment, there are no clear recommendations regarding pregnancy in climbing leaving athletes with no information about risk and benefits (93). In addition, one topic gaining more and more attention is the health of retired athletes after their active career. This also seems to be relevant for enabling (preventive) long-term and healthy participation in competitive sports (94). Overall, research should investigate topics related to the female hormonal cycle across lifespan more dominantly. Additionally, climbing associations should implement guidelines and information points to support female athletes.

## 6 Limitations

Our review has several limitations. Firstly, the unavailability of 39 full texts may have introduced bias into our results. Secondly, a similar bias may have arisen from the need to exclude a substantial number of studies that did not report the participants’ performance level. In addition, our findings are limited to advanced and higher-elite climbers and do not include results from studies on lower- to intermediate-level climbing. Whenever feasible, the study results were analyzed with respect to the participants' climbing discipline. Nevertheless, as numerous studies did not indicate the athletes' primary discipline, this should be considered when interpreting the results. Moreover, we only assessed overall male and female participation across all included studies without examining sex representation within the individual categories. Additionally geographical diversity was not addressed in this review and no studies focusing on certain competitive contexts such as speed climbing competitions were identified to address sex-related differences. Therefore, we cannot determine whether female representation is consistently low across specific research topics but can only provide a general overview. Furthermore, due to the low overall number of studies for each factor, we did not include study quality in the analysis. The quality of five studies was rated as low, with scores below 60%. Nevertheless, a full quality assessment is provided in Supplementary Table S3 to ensure transparency. Finally, due to the high heterogeneity of the studies included in the detailed analysis and the limited number of studies within the respective categories, no meta-analysis was conducted. Consequently, our results provide only a descriptive summary of the findings from the individual studies and do not present pooled effects, which should be kept in mind when interpreting the results.

## 7 Conclusion

Climbing remains an under-researched sport, particularly at the high-performance level. Additionally, although the inclusion of female athletes is steadily increasing, existing research is predominantly male-focused, both in study design and participant representation. Furthermore, we were able to identify only 34 studies conducting sex-specific analyses. Nevertheless, the results revealed notable differences between males and females in various determinants of climbing performance, such as strength, endurance, flexibility, speed, nutrition, injuries, and mental health as well as and coordination and skills. Our findings therefore emphasize the need for continued and more comprehensive research focusing on sex-specific differences and female climbing performance. Future studies should also address hormonal influences across the female lifespan and ensure detailed reporting of participant characteristics to enhance the quality, comparability, and applicability of research in this field.
