# Supplementary material for: Climbing the gap: a review on sex differences in high-level rock climbing
Source: Front Sports Act Living. 2026 Jan 9;7:1736831. doi: 10.3389/fspor.2025.1736831 (PMC12827595; doi:10.3389/fspor.2025.1736831)

Supplementary Material

**1 Supplementary Tables**

**Supplementary Table 1.** Databases and respective search-terms. No filters were used in the searching process to ensure proper transfer of search strings between databases.

| **Database** | **Search Terms and Links** |
| --- | --- |
| Pubmed | ("Mountaineering"[MeSH] OR Boulder* OR climb*) AND (performance OR training OR body OR cognition [Mesh] OR technique) AND (athlete OR sport students OR climbers) NOT stairs NOT plant NOT patient* NOT ladder |
| SURF | (Boulder* OR climb*) AND (performance OR training OR body OR cognition OR technique) AND (athlete OR sport students OR climbers) NOT stairs NOT plant NOT patient* NOT ladder |
| Web of Science (Core Collection**)** | (Boulder* OR climb*) AND (performance OR training OR body OR cognition OR technique) AND (athlete* OR sport student* OR climber*) NOT stairs NOT plant NOT patient* NOT ladder |
| APA PsychInfo | (Boulder* OR climb*) AND (performance OR training OR body OR cognition OR technique) AND (athlete* OR sport student* OR climber*) NOT stairs NOT plant NOT patient* NOT ladder |
| SPORTDiscus with full text | (Boulder* OR climb*) AND performance AND (athlete OR sport students OR climbers) NOT stairs NOT plant NOT patient* NOT ladder |
|  | (Boulder* OR climb*) AND training AND (athlete OR sport students OR climbers) NOT stairs NOT plant NOT patient* NOT ladder |
|  | (Boulder* OR climb*) AND body AND (athlete OR sport students OR climbers) NOT stairs NOT plant NOT patient* NOT ladder |
|  | (Boulder* OR climb*) AND cognit* AND (athlete OR sport students OR climbers) NOT stairs NOT plant NOT patient* NOT ladder |
|  | (Boulder* OR climb*) AND technique AND (athlete OR sport students OR climbers) NOT stairs NOT plant NOT patient* NOT ladder |

**Supplementary Table 2.** Overview and summery of all studies included in the sex-specific analysis.

| **Title** | **Author (year of publication)** | **Study aim** | **Participants** | **Discipline** | **Results regarding sex-specific differences** |
| --- | --- | --- | --- | --- | --- |
| Climbing-specific finger flexor performance and forearm muscle oxygenation in elite male and female sport climbers | Philippe et al. (2011) | To  investigate climbing-specific finger flexor strength and endurance and related muscular oxygenation in elite female and male climbers and non-climbers | female n = 12  male n = 12 | lead climbing, bouldering | Significant differences between sexes for height (*p<0.001*), body mass, body mass index (*p=0.049*), percentage of body fat mass (*p<0.001*), percentage of lean body mass (*p<0.00*1), percentage of body cell mass (*p<0.001*), maximum finger-flexor strength (*p<0.001*), strength-to-weight ratio (*p= 0.002*), and force-time integral in continuous testing  (*p=0.017*), but not for re-oxygenation and de-oxygenation during intermittent and continuous testing. Significant correlation between best on-sight climbing performance and strength-to-weight ratio (*r_2_ = 0.946, p<0.001*) for female climbers. |
| Relationship between the dietary intake of sport climbers according to climbing grading scales and the dietary supply of antioxidants | Prezeloirz, A. & Regulska-Ilow, B. (2022) | To evaluate differences in the dietary supply of selected antioxidant substances according to the climbing level | female n = 26 male n = 29 | not specified | No difference in mean energy intake between sexes with the mean energy intake being not below target. |
| Physical and Physiological Determinants of Rock Climbing | MacKenzie et al. (2019) | To identify the physical and physiological determinants of peak performance in lower-grade to elite rock climbing | female n = 33 male n = 44 | not specified | Shoulder endurance, hand and finger strength, shoulder power-endurance, hip flexibility, lower-arm grip strength, shoulder power, upper-arm strength, core-body endurance, upper-body aerobic endurance, hamstrings and lower-back flexibility, aerobic endurance, open-hand finger strength,arm span, and core-body endurance measured as maximum leg-raise hang-time significantly correlated with male climbing ability (*p<0.05*). Shoulder power and endurance, i.e. maximum pull-ups, average arm crank power, and bent-arm hang are the main determinants for climbing ability, explained a total of 77%  of the variation. Shoulder endurance and power, lower-arm grip strength, balance, aerobic endurance, and “ape index” signifcantly correlated with female climbing ability (*p<0.05*). Shoulder power and endurance explained a total of 62% of the variation. Shoulder power and endurance were found to be the main determinants of climbing ability in both sexes (*p<0.01*). No significant differences between sexes were found for cognitive or somatic anxiety , and self-confidence. After training the main determinants in males and females, climbing ability increased in both sexes. After raining variables without significant correlation to climbing ability, climbing ability did not improve in males or females. |
| Movement demands of elite female and male athletes in competitive bouldering | Medernach, J.P.,  Kleinöder, H. & Lötzerich, H.H. (2016) | To investigate the movement demands of elite female and male athletes in competitive bouldering | female n = 20 male n = 20 | bouldering | Significantly higer number  of attempts per boulder and longer rest time between attempts in females compared to males  (*p=0.008-0.04*). Significantly  higher attempt  duration  per boulder, total bouldering  time  per boulder, and average gripping time in males compared to females (*p=0.001-0.006*). |
| Dietary Intakes and the Risk of Low Energy Availability in Male and Female Advanced and Elite Rock Climbers | Monedero, J., Duff, C. & Egan, B. (2023) | To investigate the risk of low energy availability and eating disorders male and female advanced and elite rock climbers | female n = 11 male n = 14 | not specified | Significantly greater energy intake in males compared to female (*p=0.002*). Male climbers were in energy balance, while female climbers were in negative energy balance. Overall, 52% of climbers were in negative energy balance, with the prevalence of negative energy balance being 43% in male subjects and 64% in female subjects. Significantly lower EAT26 sores for males compared to females (*p=0.044*). Only females had energy intakes significantly lower than their calculated energy requirements (*p=0.006*). In all subjects, carbohydrate intakes were lower (males: *p=0.002*; females: *p<0.001*), and fat intakes were higher (males: *p<0.00*1; females: *p<0.001*) than current sports nutrition recommendations. Inadequate intakes of calcium, magnesium, and vitamin D were observed in both male and female subjects, whereas intakes of vitamins B1, B3, B12, and C were higher than the recommended values. Female subjects specifically had lower than recommended intakes of protein (*p=0.027*) and iron (*p<0.001*). |
| Nutritional Assessment, Body Composition, and Low Energy Availability in Sport Climbing Athletes of Different Genders and Categories: A Cross-Sectional Study | Mora-Fernandez et al. (2024) | To compare nutrition and body composition of climbers of different performance levels and sex | female n = 24 male n = 22 | bouldering, lead climbing, speed climbing | Significant differences between sexes were found in weight (*p<0.010*), height (*p<0.010*), and body-mass index (*p<0.010*). 54.5% and 56.5% of males and females, respectively had suboptimal energy availability values; while 31.8% and 39.1% of males and females were classified as having low-energy availability. Statistically significant sex differences were observed in the total daily energy intake (*p<0.010*), but no significant differences were observed between sexes in the estimated energy expenditure as well as in the energy intake and energy availability of athletes when expressed in relation to fat-free mass. Statistically significant differences between sexes were observed for: the total daily intake of carbohydrates (p<0.010), proteins (*p<0.01*0), and fats (*p<0.010*), but no such differences were observed when expressed in relation to the athletes’ body masses or as a percentage contribution to total energy intake. Statistically significant sex differences in another parameter were only observed when protein intake was related to athlete body mass (*p<0.050*). There were deficient intakes of all micronutrients except phosphorus in males. Statistically significant differences were observed in the daily intakes of thiamine (*p=0.011*), vitamin B6 (*p=0.046*), phosphorus (*p<0.010*), potassium (*p<0.010*), magnesium (p = 0.013), iron (p = 0.031), zinc (*p<0.010*), iodine (*p=0.011*) and selenium (*p<0.010*) between the sexes. |
| Assessment of Dietary Intake and Eating Attitudes in Recreational and Competitive Adolescent Rock Climbers: A Pilot Study | Marisa K.M., Joubert, L. & Witard, O.C. (2019) | To fill the knowledge gap in dietary intake and eating attitudes of adolescent climbers | female n = 9 male n = 13 | not specified | No significant differences between sexes for energy, carbohydrate, protein, or fat intake. Significantly higher average EAT-26 scores in males compared to females (*p=0.031*). However, these scores were not high enough to indicate high risk for disordered eating. |
| Anthropometric profiles of elite male and female competitive sport rock climbers | Watts, P.B., Martin, D.T. & Durtschi, S. (1993) | To develop descriptive anthropometric profiles of elite climbers | female n = 18 male n = 21 | lead climbing | Significantly higher climbing ability for the finalists vs semi-finalists for both sexes (*p<0.05*). Significant difference in climbing ability between male and female semi-finalists (*p<0.05*). The male semi-finalists and finalists differed in fat-free mass, whereas the female finalists were significantly shorter, lighter and had a smaller fat-free mass than the semi-finalists (*p<0.05*). Significant inter-sex differences were found for ability, height, body mass, sum of 7 skinfolds, % body fat, fat-free mass, hand volume, arm volume, grip strength, grip strength to body mass ratio (*p<0.05*) for the semi-finalists. Male finalists were significantly taller, heavier and had a greater fat-free mass, but lower % fat values, than the female finalists (*p<0.05*). No significant differences between the male semi-finalists and finalists, whereas the female finalists had significantly lower arm volume and hand grip than the female semi-finalists (*p<0.05*). Only hand volume was not significantly higher among the male finalists when compared with the female finalists. Hand volume and arm volume correlated highly with body mass (males: *r=0.54, r=0.85*; females: *r=0.75, r=0.91*). |
| Anthropometry of young competitive sport rock climbers | Watts et al. (2003) | To describe the general anthropometric characteristics of junior US competitive rock climbers | female n = 38 male n = 52 controls n = 45 | not specified | Significantly higher skinfold thickness for females compared to males climbers at  triceps, thigh, and calf (*p<0.05*). |
| Competitive performance predictors in speed climbing, bouldering, and lead climbing | Winkler, M., Künzell S. & Augste, C. (2023) | To model the influence of anthropometric components, climbing-specific power, strength and endurance parameters, flexibility, coordination, and motor planning skills on competitive climbing performance in speed, bouldering, and lead climbing | female n = 26 male n = 35 | bouldering, lead climbing, speed climbing | Significant predictors for speed climbing performance (*r^2^=44% and 35%*) were lower (*ß=.43 and .47, p<0.05*) and upper body power and strength (*ß=.40 and .37, p<0.05*) for women and men, respectively. For women’s bouldering performance (*r^2^=39%*), they were hip flexibility (*ß=.42, p<0.05*) and upper body power and strength (*ß=.37, p<0.05*), for the men’s (*r^2^*=53%) lower (ß=.41, p<0.001) and upper body power (ß=.41, p<0.001) and body fat (ß=.37, p<0.05). For women’s lead climbing (*r^2^=58%*) upper body power and strength (*ß=.59, p<0.001*) and finger endurance (*ß=.48, p<0.001*) predict performance, for the men’s (*r^2^*=58%) lower (ß=.36, p<0.05) and upper body power (ß=.28), body fat (*ß=.27*) and motor planning skills (*ß=.27*). For women, endurance did not predicts speed climbing times, finger strength was not significant in lead climbing and coordination did not enhance bouldering. For women anthropometrics and motor planning did not show predictive value in any of the disciplines. For men, strength endurance did not enhance lead climbing, body fat percentage and coordination did not play a role in speed climbing. |
| The Load Structure in International Competitive Climbing | Winkler, M., Künzell S. & Augste, C. (2022) | To characterize the external load structure of competitive climbing at an international level in the disciplines of speed, bouldering, lead, and Olympic combined | female n = 44 male n = 63 | bouldering, lead climbing, speed climbing | Significantly lower start time (*p<0.001*), number of actions of the upper limbs (*p<0.001*), number of actions of the lower limbs (*p<0.001*), contact times of the upper limbs (*p=0.023*), contact times of the lower limbs (*p=0.002*), reach times of the upper limbs (*p<0.001*) and reach times of the lower limbs (*p<0.001*) in males compared to females in speed climbing. |
| Correlations between high level sport-climbing and the development of adolescents | Schöffl et al. (2011) | To investigate anthropometric and hormonal data for climbers of the German Junior national team | female n = 13 male n = 17 | not specified | Significantly lower weight and body-mass index in girls but not in the boys, compared to the control group (*p<0.05*). Significantly greater ape index in boys compared to grils.  Significantly lower leptin values compared to calculated values and the control group in girls. |
| Evaluation of Supplement Use in Sport Climbers at Different Climbing Levels | Chmielewska, A. & Regulska-Ilow, B. (2022) | To evaluate choices of dietary supplementation, the reasons for taking them, and the source of information on supplementation among sport climbers at different levels. | female n = 40 male n = 70 | not specified | Significant differences regarding supplement usage between sexes (creatine: *p=0.03*, iron: *p=0.045*) across all performance levels. Significant differences between sees were found for hydration in  intermediate and advanced climbers. |
| The Evaluation of Energy Availability and Dietary Nutrient Intake of Sport Climbers at Different Climbing Levels | Chmielewska, A. & Regulska-Ilow, B. (2023) | To evaluate the differences in energy availability and nutrient intake of female and male sport climbers at different climbing levels | female n = 40 male n = 66 | not specified | Significant difference between energy availability in various performance levels levels in males (*p<0.001*). Significant differences between sexes for carbohydrate intake (*p=0.01*). Differences in nutrients intake accross performance levels in both males and females. |
| Performance Assessment for Rock Climbers: The International Rock Climbing Research Association Sport-Specific Test Battery | Draper et al. (2021) | To examine the validity and reliability of a battery of ten measures designed to assess the key physiological parameters for successful rock climbing performance | female n = 45 male n = 87 | not specified | Finger-flexor muscular endurance and upper-limb power explained a higher proportion of performance variance in female climbers (65%) than in males (35%). Hip-flexibility was found to distinguish between different ability levels in males but not in females. |
| Road to Paris 2024: force-velocity profile in different speed climbers' abilities | de la Cruz, V.M., Carranza, V.R. & Ravé, J.M. (2023) | To evaluate the upper and lower limbs force-velocity profile in different speed climbing abilities considering sex | female n = 11 male n = 15 | speed climbing | Significant differences between sexes were only found for maximum power in the squat with significantly higher values for the males (*p<0.05*). |
| A time motion analysis of lead climbing in the 2012 men's and women's world championship finals | Arbulu, A., Usabiaga, O. & Castellano, J. (2015) | To perform a time motion analysis of movements made by elite lead climbers and to compare results between men and women | female n = 8 male n = 8 | lead climbing | Females showed significantly greater total climbing time, frequency and duration of holds, chalk use, and rest gestures, compared to males (*p<0.05*). |
| Athletes' performance in different boulder types at international bouldering competitions | Augste, C., Sponar, P. & Winkler, M. (2021) | To analyse the occurrence of different boulder types and the athletes’ success rates in international competitions to contribute to the performance structure of competitive bouldering | women n = 20 male n = 20 | bouldering | No differences between the sexes were found in the occurrence of the different boulder types. Men solved significantly more boulder problems than women in the categories dynamo (*p<0.001*) and mantle (*p=0.014*). Women were significantly more successful with slab (*p<0.001*) problems. A significant interaction was found in the number of attempts in terms of sex and boulder type (*p<0.001*). Men needed fewer attempts for dynamos than women (*p<0.001*), but women needed fewer attempts for crimp problems (*p=0.039*) and for mantles (*p=0.008*). Lower ranked female athletes were significantly worse (*p<0.001*) than the top 20 athletes in the dynamo, volume, and crimp categories, whereas men were worse in the categories dynamo, and slab. Women among the 21+ ranked athletes accomplished significantly fewer of the boulder sections than the men among the 21+ athletes (*p=0.012*). |
| Hand-arm strength and endurance as predictors of climbing performance | Baláš et al. (2011) | To examine training characteristics, body composition, muscular strength, and endurance in sport climbers, and to demonstrate the relationship among these components by means of structural equation modelling | female n = 69 male n = 136 | not specified | Men performed better in the bent-arm hang and the grip strength test, especially in the lower performance levels. For women, relative grip strength explained more than 50% of red-point (RP) performance but less than 30% for men. Women had a higher percent body fat and demonstrated greater slope decline of the regression line than men, which means greater differences between the sexes at low levels of RP performance and less marked differences at high levels of RP performance. |
| Anthropometric and strength characteristics in young and adult elite climbers | Baláš, J., Vomácko, L. & Barbora, S. (2011) | To assess anthropometric and strength characteristics in young and adult elite climbers | female n = 24 male n = 57 | not specified | Males had significantly higher body mass, height, body fat compared to females (*p<0.001*). |
| Validity and normative scores of finger flexor strength and endurance tests estimated from a large sample of female and male climbers | Berta et al. (2025) | To address  conflicting findings regarding the validity of finger flexor strength and endurance tests in sport climbers | female n = 122 male n = 185 | not specified | Finger strength emerged as the dominant factor, explaining the majority of variance in climbing ability (males: 68%, females: 64%, *p<0.001*), followed by intermittent endurance (males: 28%, females: 34%, *p<0.001*). No significant differences were found between male and female climbers in finger flexor strength and endurance when normalized to body mass in all ability groups. |
| Mental health problems, sleep quality and overuse injuries in advanced Swedish rock-climbers - the CLIMB study | Identeg et al. (2024) | To examine the prevalence of mental health problems (depression, anxiety, and stress), sleep quality, and disability due to overuse injuries in advanced and elite rock-climbers | female n = 187 male n = 176 | not specified | A total of 30.6% of the rock-climbing group (males: 26.7, females: 35.9%) reported at least moderate levels of symptoms of depression and 23.1% (males:17.2%, females: 30.8%) at least moderate levels of symptoms of anxiety. A total of 48.4% of rock climbers (males: 39.1%, females: 61.6%) reported at least moderate levels of symptoms of stress. There were no differences observed in reported symptoms of depression, anxiety, stress or sleep quality between the rock-climbing and control group and between elite and advanced rock-climbers. |
| Prevalence of Disordered Eating Among International Sport Lead Rock Climbers | Joubert, J.M., Gonzalez, G.B. & Larson, A.J. (2020) | To assess disordered eating prevalence in rock climbers and explore the relationship between sport rock climbing ability and disorderd etating | female n = 115 male n = 383 | lead climbing | General trend towards better climbers having a lower body mass index for both females (*r=−0.329, p=0.00*) and males (*r=−0.237, p = 0.00*). Male climbers had prevalence for disordered eating of 6.3% and females of 16.5%. No significant associations were found for disordered eating and climbing ability but almost half of all females on the elite and higher-elite level showed a prevalence for disordered eating. |
| Finding new creative solutions is a key component in world-class competitive bouldering | Künzell et al. (2020) | To investigate how athletes deal with an unsuccessful first attempt of a boulder problem | female n = 15 male n = 15 | bouldering | While males change strategy after failure in 30.4% of attempts with a success rate of 22.9%, females change strategy after failure in 37.7% of attempts with a success rate of 22%. Success rate for males and females without changing the strategy after failure was 4.6 and 4.3%, respectively. |
| Heart rate and blood lactate evaluation in bouldering elite athletes | La Torre et al. (2009) | To determine blood lactate concentration and heart rate adaptations both during an official bouldering competition  and a simulated contest | female n = 5 male n = 6 | bouldering | Mean heart rate values during the recovery phases did not increase during the simulated competition in males, but slightly increased in females. |
| Attentional Differences as a Function of Rock Climbing Performance | Garrido-Palomino et al. (2020) | To investigate the relationship between attention and self-reported climbing ability while considering potential confounding factors (sex, age, climbing experience, and cardiorespiratory fitness) | female n = 10 male n = 25 | lead climbing | Males were younger, heavier, and taller than females (*p<0.05*), had a higher on-sight and red-point climbing ability with a greater cardiorespiratory fitness compared to females (*p<0.001*). No significant differences were found between males and females  for any attention tasks. |
| Role of emotional intelligence on rock climbing performance | Garrido-Palomino, I. & España-Romero, V. (2019) | To investigate the association between emotional intelligence  and self-reported climbing ability | female n = 15 male n = 28 | not specified | When adjusted for age, no significant differences between the sexes were found for "Expression and Regulation of Emotions". |
| Effect of Height on Perceived Exertion and Physiological Responses for Climbers of Differing Ability Levels | Gajdošík, J., Baláš, J. & Draper, N. (2020) | To examine differences in perceived exertion and physiological responses for climbers of different abilities completing an identical route low and high above the ground | female n = 24 male n = 18 | not specified | No significant difference between males and females for rate of perceived excertion and physiological variables except fora higher breathing frequency in females (*p=0.038*). |
| Energy and macronutrient intake of advanced polish sport climbers | Sas-Nowosielski, K. & Wycislik, J. (2019) | To assess energy and macronutrient intakes in a group of elite sport climbers | female n = 10 male n = 13 | not specified | Males were significantly taller and heavier that females (*p<0.05*). No significant differences between the sexes were found for body-mass index. In female climbers body-mass indes explained 46% of variance (*r^2^=0.46, p=0.032*) and in males 32% (*r^2^=0.32, p=0.043*). No differences between sexes was found for mean energy and macronutrients intake. Males showed a tendency towards consuming more proteins than females (*p=0.073*). |
| Fitness Profiling in Top-Level Youth Sport Climbing; Gender Differences | Vrdoljak, D., Gilić, B. & Kontić, D. (2022) | To determine gender differences in the fitness status of the top-level youth sport climbers | female n = 10 male n = 10 | not specified | No significant difference between males and females for rate of perceived excertion and physiological variables except for a significantly higher breathing frequency in females (*p=0.038*). |
| Painfully ignorant? Impact of gender and aim of training on injuries in climbing | Grønhaug et al. (2024) | To assess the distribution of chronic climbing injuries in an international population with sex-specific analyses and assess the impact of the person’s training focus or aim of training on those injuries | female n = 427 male n = 877 sex not specified n = 9 | not specified | No significant difference in the rate of injuries between sexes. Females were found to be injured less than men in the fingers (*p=0.003*), elbows (*p=0.021*) and ankle (*p=0.014*) but more in the neck (*p=0.030*) and head (*p<0.001*). In contrast, men had less chance of being injured in the shoulders than women (*p=0.001*). For the other locations, no significant differences were observed. |
| Nutrition knowledge, weight loss practices, and supplement use in senior competition climbers | Gibson-Smith et al. (2024) | To asses nutritional knowledge, weight loss for competition, and supplement use of senior competition climbing athletes. | female n = 24 male n = 26 | not specified | No significant sex differences in the general or sport nutrition knowledge scores, or effect of age. 46% percent of males and 38% of female climbers reported intentional weight loss for competition on at least one occasion. Of those, ~76% reported utilizing concerning practices, including methods that conform with disordered eating and/or eating disorders, dehydration, vomiting, and misuse of laxatives. No significant difference in supplement use between sexes. |
| Dietary Intake, Body Composition and Iron Status in Experienced and Elite Climbers | Gibson-Smith, E., Storey, R. & Ranchordas, M. (2020) | To assess dietary intake, body composition, and iron status in experienced climbers, across a range of performance levels | female n = 20 male n = 20 | not specified | 30% of male climbers and 5% of female climbers failed to meet predicted resting metabolic rate. Significantly higher energy in females compared to males when expressed relative to fat-free body mass (*p≤0.01*). No sex differences for mean carbohydrate, protein and fat intake. 30% of females met the classification criteria for iron deficiency. Significantly greater mean serum ferritin in males, compared to females (*p≤0.01*). Significant sex differences across all the measured parameters (*p≤0.05*) in body composition. Significant correlation between climbing ability and protein intake in female climbers (*r^2^= 0.452, p=0.045*). |
| Sport-specific performances in elite youth sport climbers; gender, age, and maturity specifics | Gilic, B. & Vrdoljak, D. (2023) | To investigate forearm muscle strength and determine  sex, age, and maturity status as factors of  influence on forearm muscle performance in elite youth climbers | female n = 8 male n = 10 | not specified | Body weight and body height  correlated  with  age  and  maturity  offset  in  males (*r=0.65-0.89, p<0.01*). Forearm muscle performance correlated with maturity offset in girls (*r= 0.73; p<0.05*). |

**Supplementary Table 3.** Quality of studies assessed with the AXIS instrument (Downes et al., 2016)

| **Study** | **1** | **2** | **3** | **4** | **5** | **6** | **8** | **9** | **10** | **11** | **12** | **15** | **16** | **17** | **18** | **19** | **20** | **Total** |
| --- | --- | --- | --- | --- | --- | --- | --- | --- | --- | --- | --- | --- | --- | --- | --- | --- | --- | --- |
| **Augste et al. (2021)** | 1 | 1 | 0 | 1 | 1 | 1 | 1 | 1 | 1 | 1 | 0 | 1 | 1 | 1 | 1 | 1 | 0 | **14** |
| **Balas, Pecha, et al. (2011)** | 1 | 1 | 0 | 1 | 1 | 1 | 1 | 1 | 0 | 1 | 1 | 1 | 1 | 1 | 0 | 1 | 1 | **14** |
| **Balas, Vomacko, et al. (2011)** | 1 | 1 | 0 | 1 | 0 | 1 | 1 | 1 | 1 | 0 | 1 | 1 | 1 | 1 | 0 | 0 | 0 | **11** |
| **Berta et al. (2025)** | 1 | 1 | 1 | 1 | 1 | 1 | 1 | 1 | 1 | 1 | 1 | 1 | 1 | 1 | 1 | 1 | 1 | **17** |
| **Chmielewska & Regulska-Ilow (2022)** | 1 | 1 | 0 | 1 | 0 | 1 | 1 | 1 | 1 | 1 | 1 | 1 | 1 | 1 | 1 | 1 | 1 | **15** |
| **Chmielewska & Regulska-Ilow (2023)** | 1 | 1 | 0 | 1 | 0 | 1 | 1 | 1 | 1 | 1 | 1 | 1 | 1 | 1 | 1 | 1 | 1 | **15** |
| **de la Cruz et al. (2024)** | 1 | 1 | 0 | 0 | 0 | 1 | 1 | 1 | 1 | 1 | 1 | 1 | 1 | 1 | 1 | 1 | 1 | **14** |
| **Draper et al. (2021)** | 1 | 1 | 0 | 0 | 1 | 1 | 1 | 1 | 1 | 1 | 1 | 1 | 1 | 1 | 0 | 0 | 1 | **13** |
| **Gajjdosik et al. (2020)** | 1 | 1 | 0 | 1 | 0 | 1 | 1 | 1 | 1 | 1 | 1 | 1 | 1 | 1 | 1 | 1 | 1 | **15** |
| **Garrido-Palomino & España-Romero (2019)** | 1 | 1 | 0 | 1 | 1 | 1 | 1 | 1 | 1 | 1 | 1 | 0 | 1 | 1 | 1 | 1 | 1 | **15** |
| **Garrido-Palomino et al. (2020)** | 1 | 1 | 0 | 1 | 1 | 1 | 1 | 1 | 1 | 1 | 1 | 1 | 1 | 1 | 1 | 1 | 1 | **16** |
| **Gibson-Smith et al. (2020)** | 1 | 1 | 0 | 1 | 1 | 1 | 1 | 1 | 1 | 1 | 1 | 1 | 1 | 1 | 1 | 1 | 1 | **16** |
| **Gibson-Smith et al. (2024)** | 1 | 1 | 0 | 1 | 1 | 1 | 1 | 1 | 1 | 1 | 1 | 1 | 1 | 1 | 1 | 1 | 1 | **16** |
| **Gilic & Vrdoljak (2023)** | 1 | 0 | 0 | 1 | 1 | 1 | 1 | 1 | 1 | 1 | 1 | 1 | 1 | 0 | 1 | 1 | 1 | **14** |
| **Gronhaug et al. (2024)** | 1 | 1 | 0 | 1 | 1 | 0 | 1 | 0 | 1 | 1 | 1 | 0 | 1 | 1 | 1 | 1 | 1 | **13** |
| **Identeg et al. (2024)** | 1 | 1 | 0 | 1 | 0 | 1 | 1 | 1 | 1 | 1 | 1 | 0 | 1 | 0 | 1 | 1 | 1 | **13** |
| **Joubert et al. (2020)** | 1 | 1 | 0 | 1 | 1 | 1 | 1 | 1 | 1 | 1 | 1 | 1 | 1 | 1 | 1 | 1 | 1 | **16** |
| **Künzell et al. (2020)** | 1 | 1 | 0 | 0 | 1 | 1 | 1 | 1 | 0 | 1 | 1 | 1 | 1 | 1 | 0 | 1 | 1 | **13** |
| **La Torre et al. (2009)** | 1 | 0 | 0 | 1 | 0 | 1 | 0 | 1 | 0 | 1 | 1 | 0 | 1 | 0 | 0 | 0 | 0 | **7** |
| **MacKenzie et al. (2019)** | 1 | 0 | 0 | 1 | 1 | 0 | 1 | 1 | 1 | 0 | 0 | 1 | 1 | 1 | 0 | 0 | 1 | **10** |
| **Medernach et al. (2016)** | 1 | 1 | 0 | 1 | 1 | 1 | 1 | 1 | 1 | 1 | 1 | 1 | 1 | 1 | 0 | 1 | 1 | **15** |
| **Michael et al. (2019)** | 1 | 1 | 0 | 0 | 1 | 1 | 1 | 1 | 1 | 1 | 1 | 1 | 1 | 1 | 1 | 1 | 1 | **15** |
| **Monedero et al. (2023)** | 1 | 1 | 0 | 1 | 1 | 1 | 1 | 1 | 1 | 1 | 1 | 1 | 1 | 1 | 1 | 1 | 1 | **16** |
| **Mora-Fernandez et al. (2024)** | 1 | 1 | 1 | 1 | 1 | 1 | 1 | 1 | 1 | 1 | 1 | 0 | 1 | 1 | 1 | 1 | 1 | **16** |
| **Oidui Usabiaga & Castellano (2015)** | 1 | 1 | 0 | 1 | 1 | 1 | 1 | 0 | 1 | 1 | 1 | 1 | 1 | 1 | 0 | 0 | 0 | **12** |
| **Philippe et al. (2011)** | 1 | 1 | 0 | 1 | 1 | 1 | 1 | 1 | 1 | 1 | 1 | 1 | 1 | 1 | 1 | 1 | 1 | **16** |
| **Prezeloirz & Regulska-Ilow (2022)** | 1 | 1 | 0 | 0 | 0 | 1 | 1 | 1 | 0 | 0 | 0 | 0 | 1 | 1 | 0 | 1 | 1 | **9** |
| **Sas-Nowosielski & Wycislik (2019)** | 1 | 1 | 0 | 1 | 1 | 1 | 1 | 1 | 0 | 0 | 0 | 0 | 0 | 1 | 0 | 1 | 0 | **9** |
| **Schöffl et al. (2011)** | 1 | 1 | 0 | 1 | 1 | 1 | 1 | 1 | 1 | 1 | 1 | 1 | 1 | 1 | 0 | 0 | 1 | **14** |

| **Study** | **1** | **2** | **3** | **4** | **5** | **6** | **8** | **9** | **10** | **11** | **12** | **15** | **16** | **17** | **18** | **19** | **20** | **Total** |
| --- | --- | --- | --- | --- | --- | --- | --- | --- | --- | --- | --- | --- | --- | --- | --- | --- | --- | --- |
| **Vrdoljak et al. (2022)** | 1 | 1 | 0 | 1 | 1 | 1 | 1 | 1 | 1 | 1 | 0 | 1 | 1 | 1 | 0 | 1 | 1 | **14** |
| **Watt et al. (2003)** | 1 | 1 | 0 | 1 | 1 | 1 | 1 | 1 | 1 | 1 | 1 | 1 | 1 | 1 | 0 | 1 | 1 | **15** |
| **Watts et al. (1993)** | 1 | 1 | 0 | 1 | 1 | 1 | 0 | 1 | 0 | 0 | 0 | 1 | 1 | 0 | 0 | 1 | 1 | **10** |
| **Winkler et al. (2022)** | 1 | 1 | 0 | 1 | 1 | 1 | 1 | 1 | 1 | 1 | 1 | 1 | 1 | 1 | 1 | 1 | 1 | **16** |
| **Winkler et al. (2023)** | 1 | 1 | 0 | 1 | 1 | 1 | 1 | 1 | 0 | 1 | 1 | 1 | 1 | 1 | 1 | 1 | 1 | **15** |

(1) Were the aims/objectives of the study clear?; (2) Was the study design appropriate for the stated aim(s)?; (3) Was the sample size justified?; (4) Was the target/reference population clearly defined? (Is it clear who the research was about?); (5) Was the sample frame taken from an appropriate population base so that it closely represented the target/reference population under investigation?; (6) Was the selection process likely to select subjects/participants that were representative of the target/reference population under investigation?; (8) Were the risk factor and outcome variables measured appropriate to the aims of the study?; (9) Were the risk factor and outcome variables measured correctly using instruments/measurements that had been trialled, piloted or published previously?; (10) Is it clear what was used to determined statistical significance and/or precision estimates? (eg, p values, CIs); (11) Were the methods (including statistical methods) sufficiently described to enable them to be repeated?; (12) Were the basic data adequately described?; (15) Were the results internally consistent?; (16) Were the results for the analyses described in the methods, presented?; (17) Were the authors’ discussions and conclusions justified by the results?; (18) Were the limitations of the study discussed?; (19) Were there any funding sources or conflicts of interest that may affect the authors’ interpretation of the results?; (20) Was ethical approval or consent of participants attained?

**2 Supplementary Figures**

# Supplementary Figure 1. Probability of including females as participants based on the gender of first author as well as year of publication

#
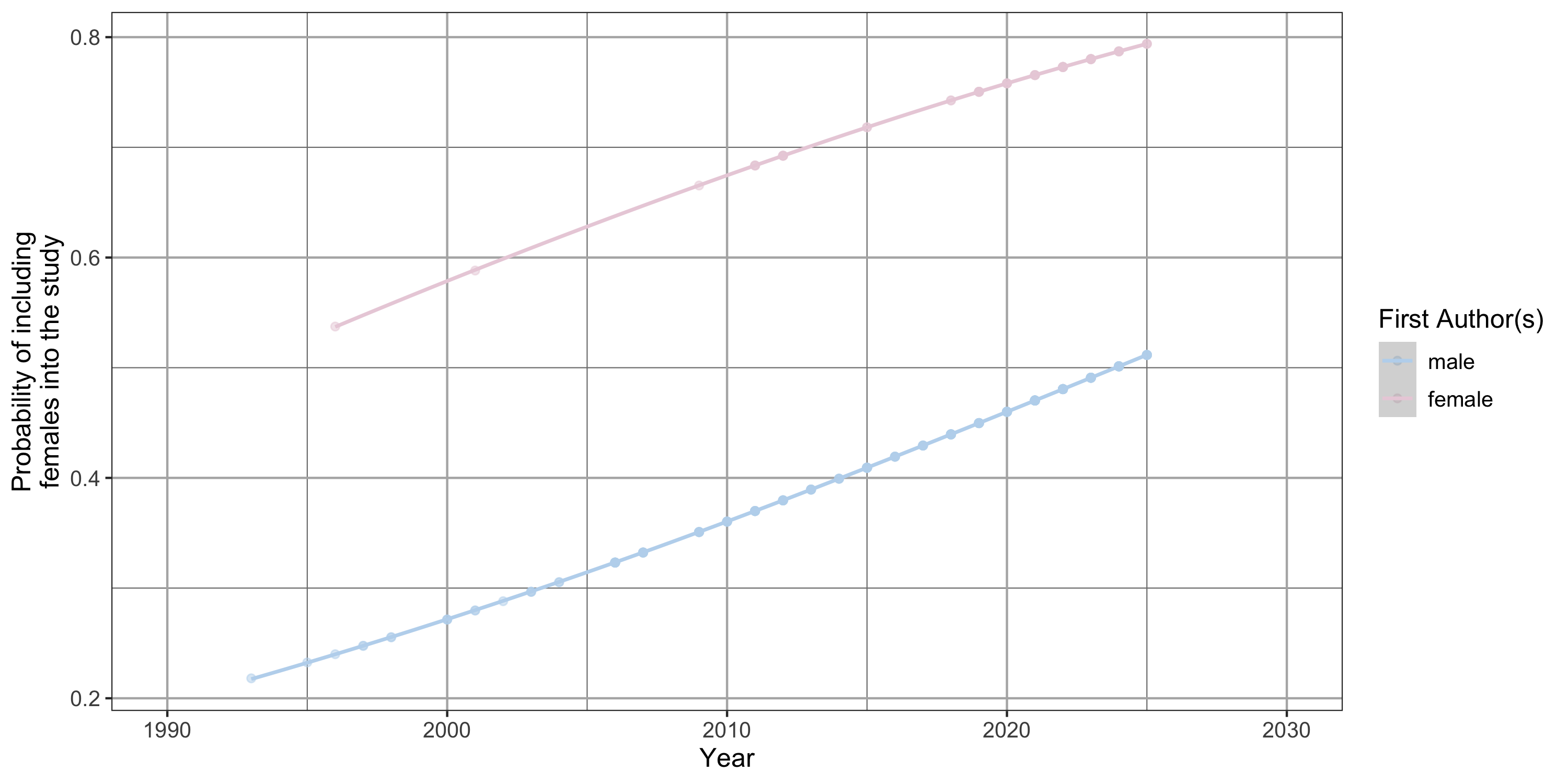

Supplement: Supplementary file 1 [file Datasheet1.docx]
